# CHOP Potentially Co-Operates with FOXO3a in Neuronal Cells to Regulate PUMA and BIM Expression in Response to ER Stress

**DOI:** 10.1371/journal.pone.0039586

**Published:** 2012-06-28

**Authors:** Arindam P. Ghosh, Barbara J. Klocke, Mary E. Ballestas, Kevin A. Roth

**Affiliations:** 1 Department of Pathology, University of Alabama at Birmingham, Birmingham, Alabama, United States of America; 2 Department of Genetics, University of Alabama at Birmingham, Birmingham, Alabama, United States of America; 3 Department of Pediatrics, University of Alabama at Birmingham, Birmingham, Alabama, United States of America; Vanderbilt University Medical Center, United States of America

## Abstract

Endoplasmic reticulum (ER) stress-induced apoptosis has been implicated in various neurodegenerative diseases including Parkinson Disease, Alzheimer Disease and Huntington Disease. PUMA (*p*53 *u*pregulated *m*odulator of *a*poptosis) and BIM (*B*CL2 *i*nteracting *m*ediator of cell death), pro-apoptotic BH3 domain-only, BCL2 family members, have previously been shown to regulate ER stress-induced cell death, but the upstream signaling pathways that regulate this response in neuronal cells are incompletely defined. Consistent with previous studies, we show that both PUMA and BIM are induced in response to ER stress in neuronal cells and that transcriptional induction of PUMA regulates ER stress-induced cell death, independent of p53. CHOP (C/EBP homologous protein also known as GADD153; gene name *Ddit3*), a critical initiator of ER stress-induced apoptosis, was found to regulate both PUMA and BIM expression in response to ER stress. We further show that CHOP knockdown prevents perturbations in the AKT (protein kinase B)/FOXO3a (forkhead box, class O, 3a) pathway in response to ER stress. CHOP co-immunoprecipitated with FOXO3a in tunicamycin treated cells, suggesting that CHOP may also regulate other pro-apoptotic signaling cascades culminating in PUMA and BIM activation and cell death. In summary, CHOP regulates the expression of multiple pro-apoptotic BH3-only molecules through multiple mechanisms, making CHOP an important therapeutic target relevant to a number of neurodegenerative conditions.

## Introduction

The ability to sense perturbations in the function of the ER is critical to eukaryotic cell survival. ER stress triggers an evolutionarily conserved intracellular response called the Unfolded Protein Response (UPR) in an attempt to restore cellular homeostasis [1]. The evolutionarily oldest branch of the UPR is triggered by the activation of a combined nuclease and kinase called IRE1 (inositol requiring protein-1). A second branch of the UPR is initiated by activation of the kinase PERK (protein kinase RNA (PKR)-like ER kinase), which similarly to IRE1, responds to ER stress by autophosphorylation and homomultimerization. A third branch of the UPR involves the protease-mediated activation of a transcriptional factor called ATF6 (activating transcription factor-6) [2]. The UPR strives to maintain ER function during stress; however, if the stress is not resolved, apoptotis is activated [3], [4]. Death inducing signals from the ER are integrated and amplified at the mitochondria and mouse embryonic fibroblasts from *Bax* ^−/−^ *Bak* ^−/−^ mice are resistant to ER stress induced-apoptosis, indicating a critical role for BAX and BAK in ER stress-triggered death [3]. Emerging evidence has implicated ER stress-induced apoptosis in a variety of chronic diseases such as diabetes, ischemia and neurodegenerative diseases [5]–[7].

Whether stressed cells live or die is largely determined by the interplay between pro-apoptotic and anti-apoptotic members of the BCL2 protein family [8]. The BH3-only proteins monitor cellular well-being and, when activated by cytotoxic signals, interact with pro-survival BCL2 relatives leading to downstream BAX and BAK activation and cell death by permeabilization of the outer mitochondrial membrane [9]. Different cell types may require different BH3-only proteins to activate apoptosis in response to the same cellular stress, while within a given cell type different BH3-only proteins may be required for activating the apoptosis machinery in response to different stimuli [10], [11]. Based on recent progress in the study of BH3-only proteins, it has become clear that they have individual differences not only in the pathways through which they are activated or induced but also in their function. The emerging diversity in the function of BH3-only proteins indicates that they are involved in more intricate molecular interplay than previously estimated, allowing them to regulate apoptosis in a more efficient manner. PUMA and BIM are the most potent of the pro-apoptotic BH3-only proteins due to their ability to bind to and neutralize all pro-survival BCL2 members [12]. Both PUMA and BIM have been implicated as key initiators of the apoptotic machinery in response to prolonged ER stress [13], [14]. BIM is essential for ER stress-induced apoptosis in a broad range of cell types, including thymocytes, macrophages and epithelial cells from breast or kidney, though different BH3-only proteins have also been implicated in this process. Gene expression profiling showed that *PUMA* and not *BIM* is transcriptionally induced in neuroblastoma cells undergoing ER stress, and RNAi-mediated suppression of PUMA protected HCT116 cells against thapsigargin-induced apoptosis, albeit not completely [13]. Alternately, PUMA as well as NOXA were reported to be critical mediators of ER stress-induced apoptosis in MEF's in a p53-dependent manner [15]. It is thus conceivable that BIM cooperates with PUMA in this apoptotic pathway and may regulate cell death to varying extents in different cell types in response to ER stress. ER stress was shown to activate BIM through two novel pathways, involving protein phosphatase 2A-mediated dephosphorylation, which prevents its ubiquitination and proteasomal degradation and CHOP-mediated direct transcriptional induction [14]. In another recent study, CHOP was shown to bind to the *Puma* promoter during ER stress and CHOP knockdown attenuated PUMA induction and neuronal apoptosis [16].

**Figure 1:**
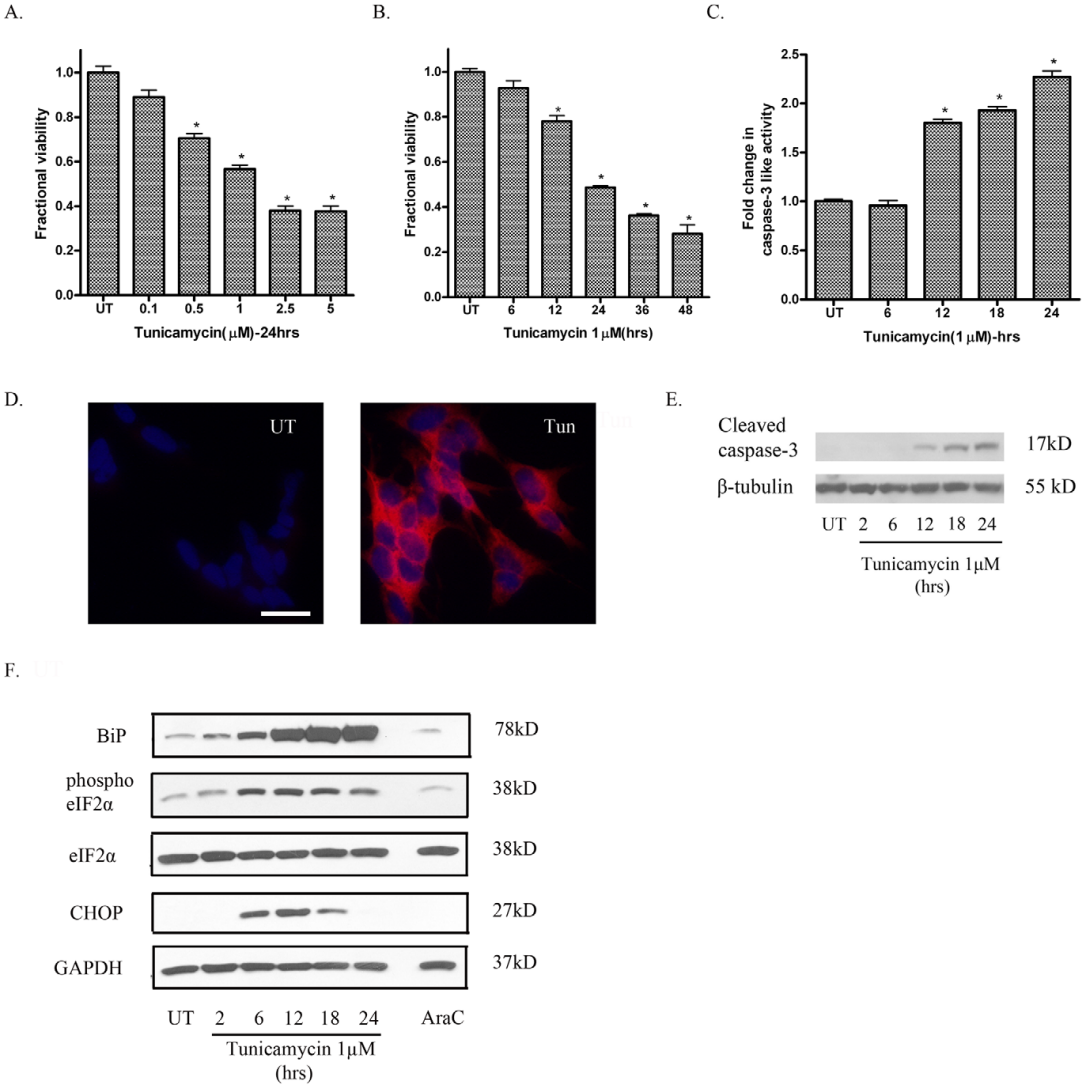
Tunicamycin produces a concentration- and time-dependent decrease in cell viability in SH-SY5Y cells concomitant with caspase-3 activation. (A) Treatment of SH-SY5Y cells with tunicamycin (0.1–5µM) for 24 h caused a progressive decrease in cell viability as measured by Calcein AM cleavage assay. (B) Treatment with tunicamycin (1µM) for 6–48 h caused a time-dependent decrease in cell viability and an increase in caspase-3 like enzymatic activity (C), as measured by the DEVD-AMC cleavage assay. (D) Treatment with tunicamycin (1µM) for 24h also produced an increase in cleaved caspase-3 immunoreactivity. (E) Treatment with tunicamycin (1µM) also produced an increase in levels of cleaved caspase-3 as measured by immunoblotting. (F) Tunicamycin (1µM) treatment of SH-SY5Y cells upregulated markers of the UPR: BiP, phospho eIF2α and CHOP on immunoblotting. Data points represent mean ± SEM, with n = 6.*p<0.05 by one-way ANOVA with Bonferroni post hoc test versus UT controls.

CHOP is thought to be the critical mediator of ER stress-induced apoptosis and studies using *Chop*-null mice have established the role of CHOP in ER stress-induced apoptosis in a number of disease models [17]–[21]. CHOP is present in the cytosol under non-stressed conditions and ER stress leads to its induction and nuclear accumulation [22]. Expression of CHOP is mainly regulated at the transcriptional level [23], though CHOP protein undergoes phosphorylation by the p38 MAP kinase family which enhances its transcriptional ability and the apoptotic response [24], [25]. FOXO3a (forkhead box, class O, 3a) belongs to the family of mammalian forkhead transcription factors, including FOXO3a (or FKHRL1), FOXO1a (or FKHR), and FOXO4a (or AFX), which are regulated by growth factor receptor-induced activation of the phosphatidylinositol 3-kinase (PI3K)/AKT (or protein kinase B) signaling pathway [26]–[28]. Activation of AKT causes phosphorylation of forkhead transcription factors, in the case of FOXO3a at the threonine32, serine253, and serine315 sites [26]. Phosphorylation of FOXO3a promotes its redistribution from the nucleus to the cytosol and therefore reduces its DNA binding and transcriptional activity [29]. Studies in mammalian cells have shown that activation of FOXO3a by decreasing its phosphorylation and increasing its nuclear content can stimulate the expression of proteins that are involved in either apoptosis [26] or cell cycle arrest [30] in different types of cells. FOXO3a is an important regulator of BIM expression in neurons in response to NGF withdrawal [31] and alterations in the AKT-FOXO3a axis have been reported to affect BIM expression in neurons in response to hypoxia-ischemia [32] or ER stress [33]. FOXO3a has also emerged as a key transcriptional regulator of PUMA expression in response to cytokine withdrawal [34] and other cytotoxic stimuli [35], [36].

**Figure 2:**
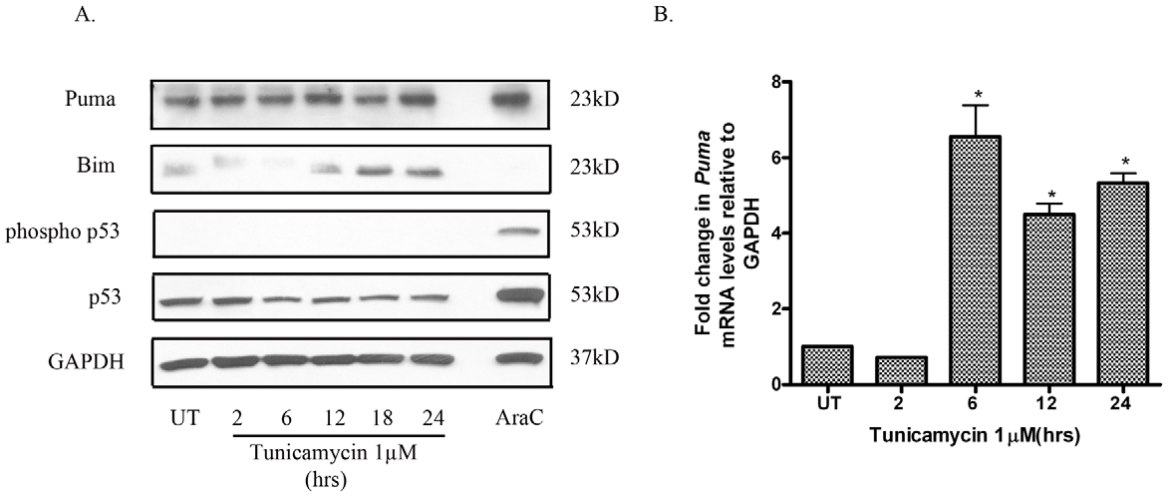
ER stress in neuronal cells induces the expression of pro-apoptotic proteins PUMA and BIM. (A) Tunicamycin (1µM) induces a time-dependent increase in PUMA and BIM protein levels. Though AraC caused an increase in PUMA and phospho p53 protein levels, tunicamycin-induced increase in PUMA protein levels was not associated with an increase in phospho p53 levels. (B) Tunicamycin treatment also caused a robust increase in *Puma* mRNA levels as measured by quantitative PCR. Data points represent mean ± SEM, with *n* = 3.*p<0.01 by one-way ANOVA/Bonferroni post hoc test versus UT controls.

In the present study, we examined the role of CHOP in mediating the induction of PUMA and BIM in response to ER stress in neuronal cells and assessed the potential interaction of CHOP with FOXO3a in an attempt to gain further mechanistic insights linking CHOP to the activation of pro-apoptotic BH3-only family members.

**Figure 3:**
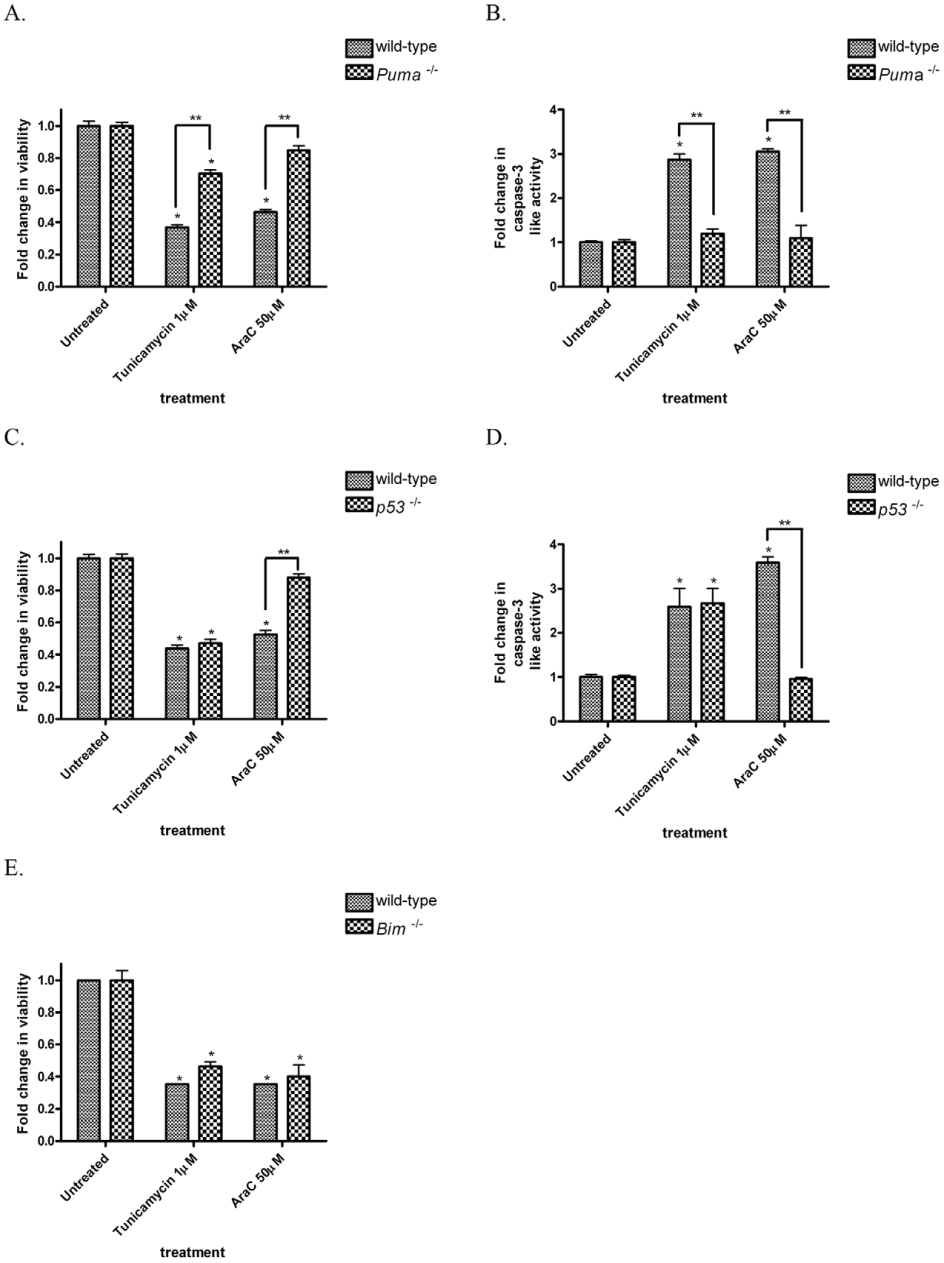
Loss of *Puma* but not *p53*, protects against tunicamycin-induced cell death and caspase-3 activation. (A) PUMA-deficient telencephalic neurons exhibited significantly less death after exposure to tunicamycin in comparison to wild-type telencephalic neurons from litter mate controls. Similarly, PUMA-deficient telencephalic neurons were protected against AraC-induced cell death (B) PUMA-deficient telencephalic neurons exhibited a significant attenuation in caspase-3 activation after exposure to tunicamycin or AraC in comparison to wild-type neurons. (C) p53-deficient telencephalic neurons did not exhibit significant protection against tunicamycin-induced cell death although they were protected from AraC-induced cell death in comparison to wild-type telencephalic neurons. (D) p53-deficient telencephalic neurons exhibited a significant attenuation in caspase-3 activation after exposure to AraC in comparison to wild-type litter control neurons but not after treatment with tunicamycin. (E) BIM-deficient telencephalic neurons demonstrated no significant protection against tunicamycin-induced cell death in comparison to wild-type littermates. The data represent mean ± SEM, with n = 5. *p<0.01 by two-way ANOVA/Bonferroni post hoc test compared to both the wild-type and the knock-out treated group.

## Materials and Methods

### Mice

The generation of mice deficient for *Puma* and *Bim* have been previously described [37], [38]. *p53* ^−/−^ mice were purchased from Taconic (Germantown, NY). All mice were backcrossed for at least 6 generations onto the C57BL/6J background. The morning on which a vaginal plug was seen was designated as embryonic Day 0.5 (E0.5). Pregnant mice were anesthetized with methoxyflurane and sacrificed on gestational day 14 by cervical dislocation. Embryos (E14.5) were removed for generation of telencephalic neuronal cultures and tail and limb samples were taken for DNA extraction and PCR analyses. Mice were housed and cared for according to the *NIH Guide for the Care and Use of Laboratory Animals* and the Institutional Animal Care Committee of the University of Alabama at Birmingham. All animal protocols were approved by the Institutional Animal Care and Use Committee of the University of Alabama at Birmingham.

**Figure 4:**
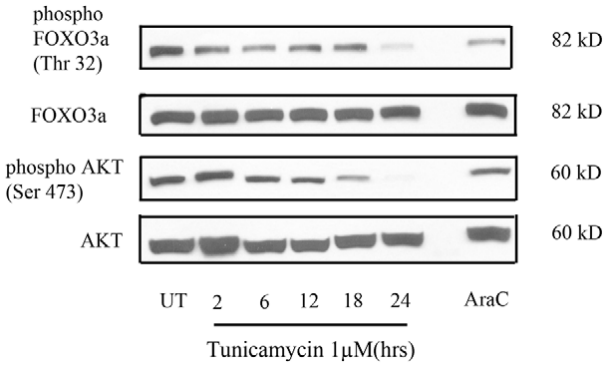
ER stress causes a change in the AKT/FOXO3a axis. Tunicamycin (1µM) induced a progressive dephosphorylation of AKT (Ser 473) accompanied by a progressive dephosphorylation of FOXO3a (Thr 32). In comparison to ER stress, genotoxic stress does not induce changes in phospho AKT or phospho FOXO3a levels.

### Primary telencephalic cell cultures

E14.5 telencephalic cells were dissociated as previously described [39] and plated in a chemically defined serum-free medium containing insulin, transferrin, selenium, progesterone, putrescine, glucose and glutamine [39] followed by incubation at 37°C in humidified 5% CO_2_/95% air atmosphere for 48 h prior to treatment. Cells were plated at 30,000 cells/well in 48 well plates coated with poly-L-lysine and laminin. Cultured cells were treated with tunicamycin or cytosine arabinoside (AraC) (Sigma St. Louis, MO, USA) for 24h to measure cell viability and caspase-activation.

**Figure 5:**
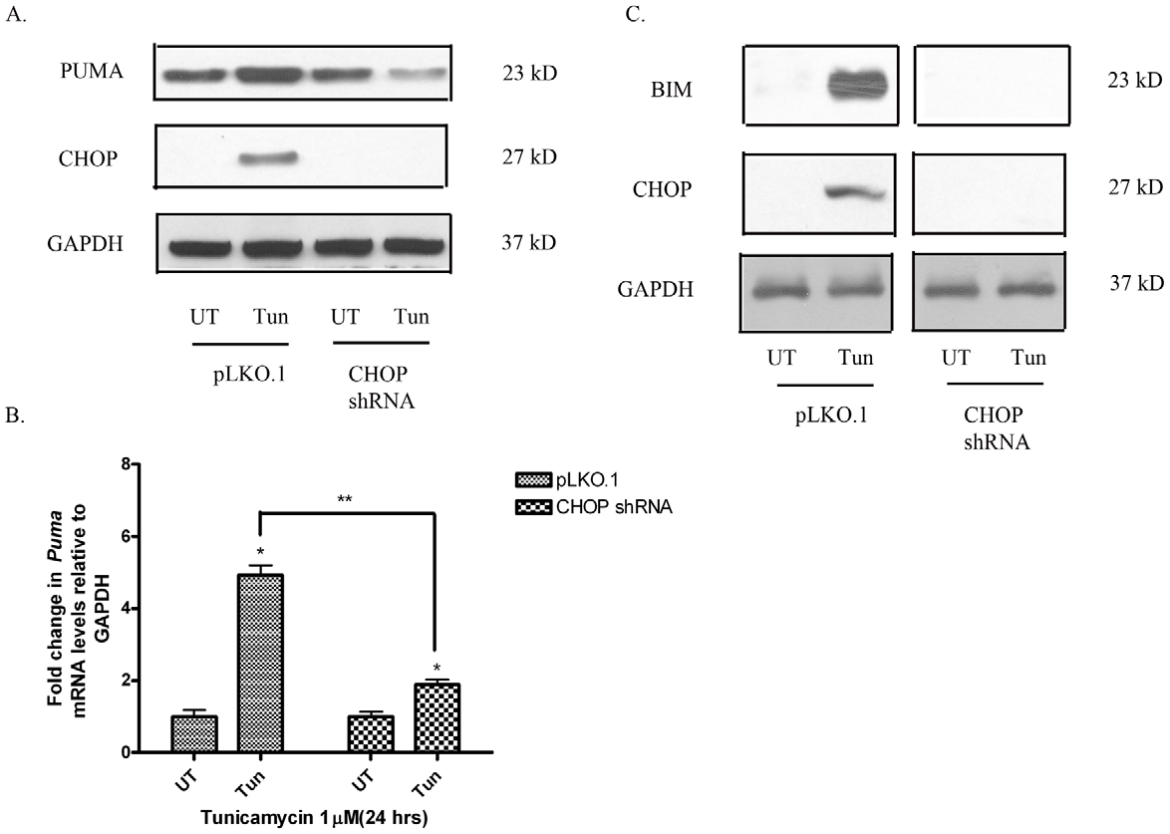
CHOP knockdown inhibits the induction of PUMA and BIM in neuronal cells in response to ER stress. (A) Knockdown of CHOP in SH-SY5Y cells using shRNA ameliorated the induction of PUMA with tunicamycin in comparison to control cells. (B) Knockdown of CHOP also attenuates the transcriptional induction of *Puma* mRNA in comparison to controls. (C) Knockdown of CHOP also causes a decrease in the activation of BIM in comparison to control cells after treatment with tunicamycin. Data points represent mean ± SEM, with n = 3. *p<0.05 by 2-way ANOVA/Bonferroni post hoc test vs. treated controls and **p<0.05 by 2-way ANOVA/Bonferroni post hoc test vs. treated CHOP knockdowns.

### Cells

SH-SY5Y cells were obtained from ATCC (CRL-2266) and cultured in Minimum Essential Medium Eagle (Cellgro, Herndon, VA, USA) and F12-K (ATCC, Manassas, VA, USA) medium supplemented with 0.5% sodium pyruvate (Cellgro), 0.5% non-essential amino acids (Cellgro), 1% penicillin/streptomycin (Sigma), and 10% fetal bovine serum (FBS; HyClone, Logan, UT, USA). Cells were plated at a density of 30,000 cells per well in 48 well plates and grown for 24 h prior to treatment. Cells were treated with tunicamycin (1µM for 0–24h) or AraC (50µM for 24h) for all experiments.

**Figure 6:**
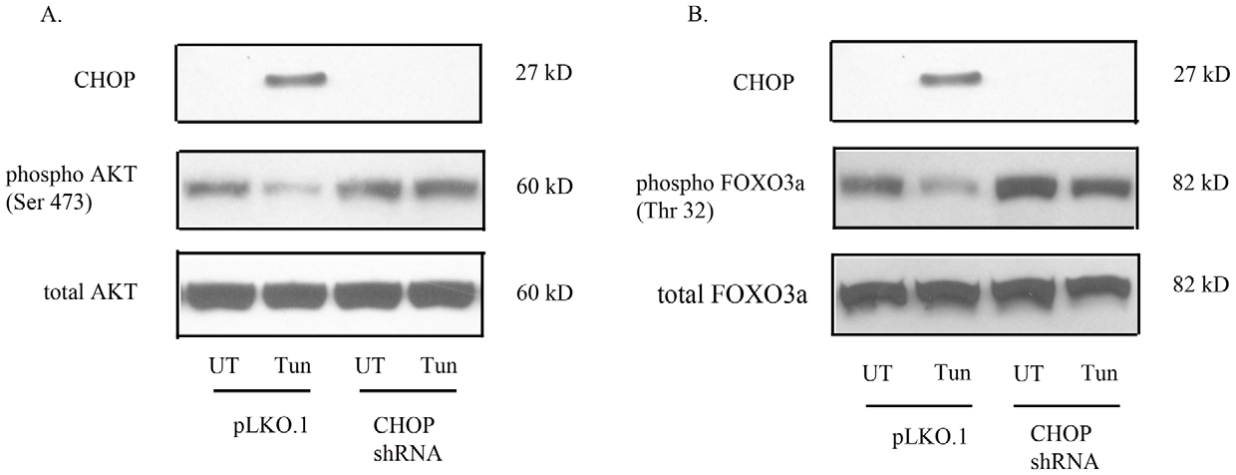
CHOP knockdown prevents dephosphorylation of AKT and FOXO3a. (A) CHOP knockdown prevents dephosphorylation of AKT (Ser 473) in comparison to control cells treated with tunicamycin. (B) CHOP knockdown prevents dephosphorylation of FOXO3a (Thr 32) in comparison to control cells treated with tunicamycin.

### Cell Viability and Caspase Assays

Cell viability in SH-SY5Y cells and primary telencephalic neurons was assessed by the Calcein AM assay. Briefly, cells were washed in Locke's buffer (154 mM NaCl, 5.6 mM KCl, 3.6 mM NaHCO_3_, 2.3 mM CaCl_2_, 1.2 mM MgCl_2_, 5.6 mM glucose, 5 mM HEPES, pH 7.4). 5 μM Calcein AM (Molecular Probes, Eugene, OR) was diluted in this buffer and added to cells; they were then incubated at 37°C for 30 minutes. Calcein AM conversion was measured using a fluorescence plate reader (excitation 488 nM, emission 530 nM). To assess caspase activity in vitro, we utilized the DEVD-AMC labeled caspase substrate cleavage assay. Following treatment, cells were lysed in 100μl buffer A (10 mM HEPES, pH 7.4, 42 mM KCl, 5 mM MgCl_2_, 1 mM DTT, 0.5% CHAPS, 10% sucrose, 1 mM PMSF, and 1μg/ml leupeptin) followed by 150 μl of buffer B (25 mM HEPES, pH 7.4, 1 mM EDTA, 0.1% CHAPS, 10% sucrose, and 3 mM DTT) containing 10 μM DEVD-AMC (Biomol, Plymouth Meeting, PA) and incubated at 37°C for 30 minutes. Production of the fluorescent AMC caspase-3 product was measured with a fluorescence plate reader (excitation 360 nM, emission 460 nM). Both assays were expressed in comparison to untreated controls.

**Figure 7:**
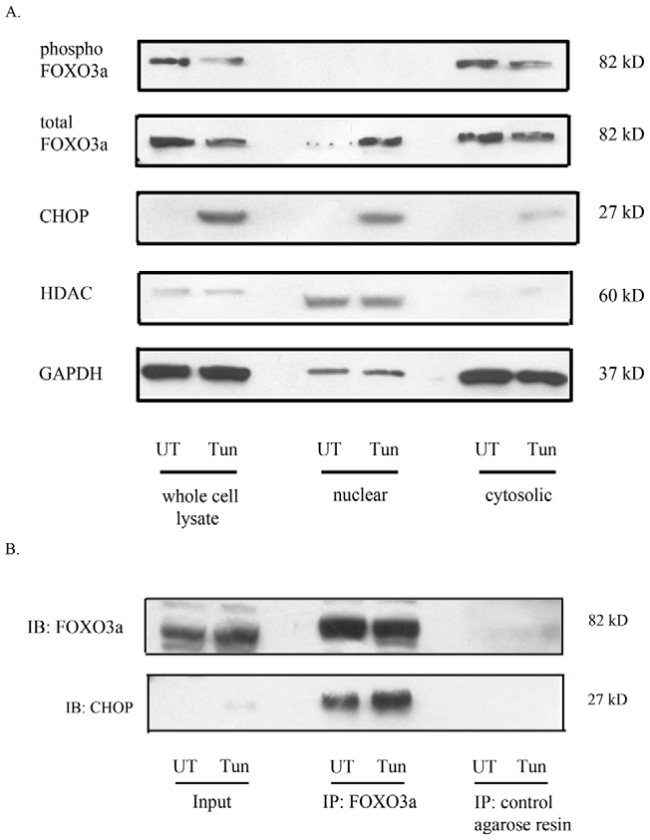
CHOP directly interacts with FOXO3a in response to ER stress. (A) Nuclear and cytoplasmic fractions from untreated SH-SY5Y cells and those treated with tunicamycin (1µM) for 24h were examined for levels of total and phosphorylated FOXO3a and CHOP. GAPDH was used as a loading control for whole cell lysates or cytoplasmic fractions, while HDAC was used as a loading control for nuclear fractions (B) Cell lysates of SH-SY5Y cells treated with tunicamycin or controls were subjected to immunoprecipitation using FOXO3a polyclonal antibody or control IgG. CHOP protein was detected from immunoprecipitates by western blotting in comparison to input controls.

### Immunocytochemistry

Cells were fixed in 4% paraformaldehyde for 20 minutes at 4°C followed by phosphate-buffered saline (PBS) wash and stored at 4°C. Cells were permeabilized with PBS-blocking buffer (PBS with 0.1% BSA, 0.3% Triton X-100, and 0.2% nonfat powdered milk) for 30 minutes at room temperature (RT). The primary antibody against cleaved caspase-3 (# 9661, Cell Signaling Technologies, Danvers, MA) was incubated overnight at 4°C in PBS blocking buffer without Triton. Plates were washed with PBS and a horseradish peroxidase-conjugated horse anti-rabbit SuperPicture (Zymed Laboratories Inc., South San Francisco, CA) secondary antibody was applied for 1 hour at RT. Plates were washed with PBS and immunoreactivity was detected using Tyramide Signal Amplification (TSA) system (Perkin-Elmer Life Science Products, Boston, MA). Following PBS washes the cells were counterstained with bisbenzimide (2 μg/ml; Hoechst 33258; Sigma) and examined with a Zeiss Axioskop microscope equipped with epifluorescence. Images were captured using Axiovision® software.

**Figure 8:**
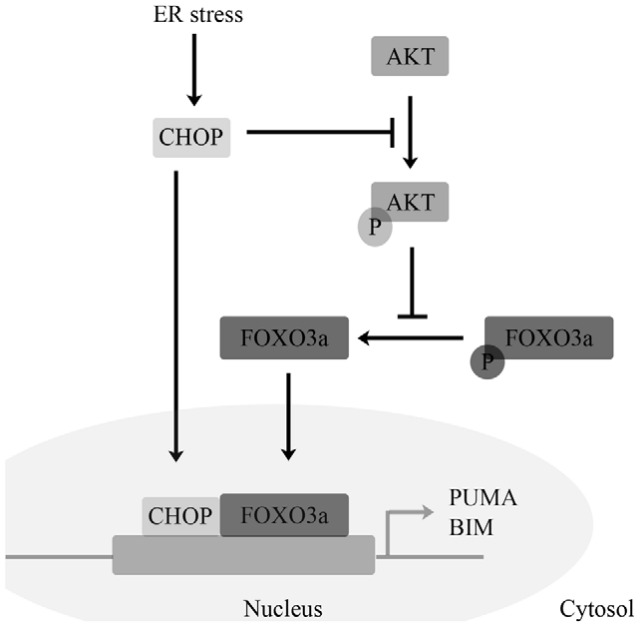
Proposed model for direct and indirect activation of PUMA and BIM by CHOP.

### RNA extraction and Real Time PCR analysis

Cells were treated with tunicamycin at various time points or with AraC and total RNA was extracted using the RNeasy Plus minikit (Qiagen; Maryland, USA) according to the manufacturer's instructions. Real-time RT-PCR to measure Puma mRNA was performed using TaqMan One-Step RT-PCR master mix reagents (catalog no. 4309169; Applied Biosystems) and a Taqman Gene Expression Assay (assay ID: Hs00248075_m1) using the manufacturer's handbook as a reference. Glyceraldehyde 3-phosphate dehydrogenase (GAPDH; assay ID: Hs99999905_m1) was studied in parallel as an internal control. TaqMan RT-PCR reactions were performed in 25-µl final volumes containing 5 µl (10x dilution of stock) of RNA sample, AmpErase UNG (2x, 12.5 µl), MultiScribe reverse transcriptase and RNase inhibitor (40x, 0.625 µl), primers and probe (20x, 1.25 µl), and RNase-free water (5.625 µl). Quantitative real-time PCR was performed using the ABI PRISM 7500 sequence detection system. Five 1-log serial dilution reactions were conducted in duplicate. Data were exported from ABI PRISM 7500 software into Microsoft Excel and analyzed using the relative standard curve method. Cycle threshold values for each serial dilution were plotted, and the values were calculated from the y-intercept and slope of the standard curve using the Excel Trendline option. These values were then used to calculate the input amount of mRNA samples. The input amount of target mRNA was normalized to GAPDH mRNA as an endogenous control.

### RNAi

Lentiviral shRNA (CHOP) constructs were purchased from Open Biosystems (RHS4533-NM_004083). shRNAs were cotransfected into 293FT cells together with packaging plasmids by following the manufacturer's protocol (Invitrogen ViraPower^TM^ Lentiviral Expression Systems kit, Carlsbad, CA). The pLKO.1 lentivirus plasmid vector without a shRNA insert was used as a negative control in experiments using the shRNA constructs.SH-SY5Y cells were passaged and plated in a 6-well plate and allowed to adhere for 24 h before infection. SH-SY5Y cells were transduced (MOI = 10) in the presence of polybrene overnight, and the following day media were replaced by fresh media. After 24 h cells were selected by treating with media containing 1.5μg/ml puromycin. Protein levels and experiments were assessed after 72 h.

### Preparation of cell lysates

Preparation of whole cell lysates was performed as follows. Briefly, cells were lysed by adding ice cold RIPA buffer (50 mM Tris, pH 8.0, 150 mM NaCl, 0.5% sodium deoxycholate, 0.1% SDS, and 1.0% NP-40) supplemented with with 1% PMSF, 1% protease inhibitor cocktail (Sigma) and 1% phosphatase inhibitor cocktails (Sigma). Lysates were subject to constant agitation for 30 minutes at 4°C, sonicated and centrifuged at 12000g for 20 minutes to pellet out the cell debris. The supernatant was transferred to a fresh tube, and the protein concentrations were determined via Pierce BCA assay kit (Pierce). Nuclear–cytoplasmic fractionation was performed using the NE-PER Nuclear and Cytoplasmic Extraction Reagents kit (Thermo Fisher Scientific) according to the manufacturer's protocol and the protein concentrations were determined via Pierce BCA assay kit (Pierce).

### SDS-PAGE and Western blotting

Equal amounts of whole cell lysates (35 μg), and nuclear or cytoplasmic fractions (20µg) were resolved by SDS-PAGE and transferred to PVDF membranes. Blots were blocked for 1 h at room temperature, 5% milk in wash buffer (200 mm Tris base, 1.37 M NaCl, 1% Tween 20, pH 7.6) followed by overnight incubation with primary antibodies. Blots were probed for either p53 (2524, Cell Signaling), phosho-p53 (9284, Cell Signaling), Akt (9272, Cell Signaling), phospho-Akt (9271, Cell Signaling), Bip (3177, Cell Signaling), CHOP (2895, Cell Signaling), eIF2α (2103, Cell Signaling), phospho-eIF2α ( 3398, Cell Signaling), Akt (9272, Cell Signaling), phospho- Akt (9271, Cell Signaling), phospho-FoxO3a (2599, Cell Signaling) and cleaved caspase-3 (9661, Cell Signaling), Puma (sc-19187, Santa Cruz), Bim ( B7929, Sigma) and FoxO3a( 07-702, Millipore) with GAPDH (2118, Cell Signaling) or HDAC (H-51, Santacruz Biotechnology) serving as a loading control. After primary antibody incubation, all blots were washed with 1× Tris-buffered saline containing 0.1% Tween 20, then incubated with appropriate secondary antibodies, for 1 h at room temperature and washed. Signal was detected using ECL (Pierce) chemiluminescence.

### Co-Immunoprecipitation

Co-immunoprecipitation experiments were performed using the Pierce Co-Immunoprecipitation Kit (26149, Pierce; Rockford, IL) as per the manufacturer's instructions. Briefly, 200µg of protein lysates were pre-cleared using a control agarose resin to minimize non specific binding. These lysates were then applied to columns containing 1µg immobilized antibodies (FoxO3a or CHOP) covalently linked to an amine-active resin and incubated overnight at 4°C. Equal volumes of the lysates were also applied to columns containing control resin and processed the same as the antibody coupling resin for negative controls. The co-immunoprecipitate was then eluted and analyzed by SDS-PAGE along with the input controls.

### Statistics

For experiments involving quantification, mean and SEM were determined from at least 3 independent experiments with an “n” of one representing one gene disrupted mouse accompanied by one wild-type litter mate control or separate experiments from different SH-SY5Y passages. Effects of genotype for each age were analyzed for significance using one-way or two-way ANOVA, followed by Bonferroni test for all pair-wise comparisons. In all cases, a p value of less than or equal to 0.05 was considered significant.

## Results

### Tunicamycin induces apoptosis and an increase in markers of ER stress in SH-SY5Y cells

Microscopic examination of SH-SY5Y cells treated with tunicamycin (a protein N-glycosylation inhibitor and well characterized ER stress inducing agent) [40], revealed cell shrinkage and gradual cell detachment from culture dishes (data not shown). Tunicamycin induced a concentration-(Fig. 1a) and time-dependent (Fig. 1b) decrease in cell viability in SH-SY5Y cells as measured by the Calcein-AM conversion assay. The decrease in cell viability with tunicamycin treatment was accompanied by a concomitant increase in caspase-3 like enzymatic activity as measured by the DEVD-AMC cleavage assay (Fig. 1c) There was also an increase in cleaved caspase-3 immunoreactivity (Fig. 1d.) and an increase in cleaved caspase-3 protein levels by western blot analysis (Fig. 1e). The induction of ER stress in SH-SH5Y cells after treatment with tunicamycin was indicated by a robust increase in the levels of BIP/GRP78 (Fig. 1f). We also observed an increase in phosphorylated eIF2α levels suggesting that the upstream arms of the UPR were being reliably activated (Fig. 1f). In addition to an increase in the markers of the UPR, we observed a time-dependent increase in the levels of CHOP, suggesting that ER stress-dependent apoptotic pathways were being engaged in response to prolonged tunicamycin exposure (Fig. 1f). To test the stimulus-specific effects of tunicamycin on neuronal cells, we compared it with AraC, a known genotoxic agent that causes cell death through a p53- and PUMA-dependent mechanism [41]. In contrast to tunicamycin treated cells, treatment of SH-SY5Y cells with AraC did not cause an increase in markers of the UPR or an increase in levels of CHOP (Fig. 1f).

### ER stress causes an induction of BH3-only proteins PUMA and BIM

*Puma* and *Bim* are two potent BH3-only genes whose expression is known to be robustly increased in response to ER stress [13], [14]. Consistent with previous reports, we detected an increase in PUMA and BIM protein levels in response to tunicamycin (Fig. 2a). *Puma* is a direct transcriptional target of the tumor suppressor p53, which is activated by DNA damage, oncogene-induced stress and certain other cytotoxic stimuli [37], [42]. Previous studies have shown that ER stress-induced PUMA expression is largely indepdendent of p53 [13]. We also noted a significant increase in *Puma* mRNA levels in SH-SH5Y cells treated with tunicamycin (Fig. 2b) and consistent with previous reports, we did not observe changes in the levels of phosphorylated or total p53 in SH-SY5Y cells in response to tunicamycin in comparison to cells treated with AraC (Fig. 2a).

### PUMA deficiency significantly protects neurons against ER stress induced-cell death and caspase activation

To assess the differential roles of PUMA, BIM and p53 in the regulation of ER stress-induced neuronal cell death, we generated primary telencephalic neuron cultures from *Puma* ^−/−^, *Bim* ^−/−^ and *p53* ^−/−^ E14.5 mice and treated them with tunicamycin or AraC, and compared them to cultures prepared from wild-type littermate controls. PUMA-deficient telencephalic neurons were significantly protected from tunicamycin-induced cell death and caspase-3 activation in comparison to wild-type cells (Fig. 3a and 3b). As we have previously reported, PUMA deficiency also afforded significant protection against genotoxic stress-induced apoptosis and caspase-3 activation (Fig. 3a and 3b). Though p53 deficiency attenuated AraC-induced apoptosis and caspase-3 activation, it did not protect against tunicamycin-induced cell death or caspase-3 activation (Fig. 3c and 3d). Though we observed a robust induction of BIM in SH-SY5Y cells in response to tunicamycin, BIM-deficient telencephalic neurons were not significantly protected against tunicamycin- or AraC-induced cell death in comparison to wild-type littermate controls (Fig. 3e).

### ER stress in SH-SY5Y cells activates the AKT-FOXO3a axis

To assess the potential role of the AKT-FOXO3a axis in regulating both PUMA and BIM expression in response to ER stress, we examined the levels of phosphorylated AKT (serine 473) and its downstream target phosphorylated FOXO3a (threonine 32) in SH-SY5Y cells in response to tunicamycin. As reported previously in neuronal cells [33], tunicamycin treatment caused a time-dependent decrease in the levels of phosphorylated AKT and a decrease in phosphorylated FOXO3a (Fig.4), suggesting an activation of the AKT-FOXO3a axis in response to ER stress. In contrast, treatment of cells with AraC did not induce dephosphorylation of AKT or FOXO3a, indicating that the activation of this axis was specific to the induction of ER stress (Fig. 4).

### Induction of Puma and Bim in response to ER stress is CHOP-dependent

To assess the role of CHOP in inducing the BH3-only molecules PUMA and BIM in response to ER stress, SH-SY5Y cells transduced with pLKO.1 and CHOP shRNA were treated with tunicamycin for 24h and compared to untreated controls. CHOP knockdown resulted in a significant decrease in PUMA protein (Fig. 5a) and *Puma* mRNA levels (Fig. 5b). As reported previously, knockdown of CHOP expression led to a significant attenuation in BIM protein levels in response to tunicamycin treatment (Fig. 5c). These results suggest that CHOP directly regulates the expression of pro-apoptotic proteins BIM and PUMA in neuronal cells in response to ER stress.

### Dephosphorylation of Akt and FOXO3a in response to ER stress is CHOP-dependent

To determine whether ER stress inactivated AKT in a CHOP-dependent manner, the expression of CHOP was selectively inhibited by shRNA. Knockdown of CHOP prevented the dephosphorylation of AKT (Ser473) induced by tunicamycin (Fig. 6a). Knockdown of CHOP also prevented the dephosphorylation of its downstream target FOXO3a (Thr32) (Fig. 6b). These results suggest that CHOP may play a key role in regulating other putative transcriptional regulators of BIM and PUMA expression in neurons in response to ER stress.

### CHOP interacts with FOXO3a in neuronal cells in response to ER stress

ER stress causes an induction of the pro-apoptotic transcription factor CHOP that then activates its various downstream targets. Similarly, as previously reported [33] FOXO3a undergoes dephosphorylation and nuclear translocation in neuronal cells in response to ER stress in a PI3K-AKT dependent manner. To assess the localization of CHOP and FOXO3a in response to ER stress, we performed nuclear and cytosolic fractionation studies and assessed the levels of CHOP and total FOXO3a in these fractions. Consistent with previous reports, we found an increase in CHOP protein levels in the nuclear fraction after treatment with tunicamycin. Similarly, we observed an increase in the levels of FOXO3a in the nuclear fraction after treatment with tunicamycin, while the transcriptionally inactive, phosphorylated FOXO3a remained in the cytosolic fraction (Fig. 7a). Thus our results suggest alterations in the nuclear fractions of the key transcription factors CHOP and FOXO3a in response to tunicamycin treatment. To test for a functional interaction between CHOP and FOXO3a in response to ER stress, we performed a co-immunoprecipitation experiment. Protein extracts from untreated and tunicamycin treated cells were subjected to Co-IP with an antibody against FOXO3a or control resin, and subsequently analyzed through Western blotting with an anti-CHOP polyclonal antibody. Representative results demonstrate that CHOP was co-precipitated with the anti-FOXO3a antibody but not with the control resin in comparison to the input controls (Fig. 7b).

## Discussion

Increasing evidence points to the role of ER stress in the pathophysiology of various neurodegenerative diseases. Aberrant aggregated proteins and elevation of markers of ER stress have been observed in dying neurons in animal models of ischemia [43], Parkinson disease [20], Huntington disease [44] and Alzheimer disease [45]. It is thus essential to delineate the mechanisms by which ER stress can induce apoptosis in neuronal cells. Our current results support previous studies indicating that ER stress in neuronal cells upregulates key pro-apoptotic molecules PUMA and BIM, and that PUMA critically regulates ER stress-induced neuronal apoptosis. Loss of CHOP expression attenuated PUMA and BIM induction in response to ER stress in neuronal cells. Our results also indicate that CHOP may potentially interact with FOXO3a, a common upstream transcriptional regulator of *Puma* and *Bim* expression in neuronal cells in response to ER stress (Fig. 8), though further knockdown studies are needed in support of our preliminary data and to determine the functional significance of this interaction. These findings highlight the complex roles of CHOP in mediating ER stress-induced apoptosis by transcriptional mechanisms and also by mediating protein-protein interactions.

The BH3-only proteins sense and relay stress signals, and are activated in a cell-type- and stimulus-dependent manner [9]. The BH3-only proteins activate BAX and BAK directly or indirectly, by engaging and neutralizing their pro-survival relatives, which otherwise constrain BAX and BAK from causing mitochondrial outer membrane permeabilization [8]. PUMA and BIM have been implicated in regulating apoptosis in response to ER stress [14], [16], [46] and consistent with previous studies, we show that PUMA and BIM are both induced in neuronal cells in response to ER stress, though the transcriptional induction of *PUMA* alone is critical for the apoptotic response. We and others have previously demonstrated that the induction of PUMA in response to genotoxic stress is mediated by p53 [41], [47]. However, we did not observe changes in upstream p53 signaling in response to ER stress in neuronal cells, and p53-deficient telencephalic neurons were not protected against tunicamycin-induced cell death or caspase-3 activation suggesting that PUMA-dependent ER stress-induced death occurs independent of p53 expression.

The most significant ER stress-induced apoptotic pathway is mediated through CHOP. CHOP/GADD153 is a bZiP transcription factor that is induced through the ATF6 and PERK UPR pathways [22], [48]. One of the more widely cited mechanisms of CHOP-induced apoptosis is suppression of the pro-survival protein BCL2 [49]. Other CHOP-induced molecules that have been implicated in apoptosis include death receptor-5 (DR5; TRAIL-R2) and Tribbles-related protein 3 (TRB3). A previous study using multiple ER stressors demonstrated the importance of BIM in ER-stress-induced apoptosis in some cell types but not others. ER stress increased BIM levels through both decreased proteasomal degradation and CHOP C/EBPα-mediated gene induction [14]. Our results indicate that CHOP regulates expression of both PUMA and BIM in neuronal cells in response to ER stress, but that PUMA plays a more significant role than BIM in mediating death. Although our study clearly demonstrates the important involvement of CHOP signaling in ER-stress-induced neuronal apoptosis, it is evident that inhibition of this pathway does not completely abrogate the transcriptional induction of PUMA or cell death. This suggests that additional transcriptional factors may contribute to PUMA activation during ER-stress-induced apoptosis or PUMA may co-operate with other BH3-only proteins such as BIM and BID to mediate the full apoptotic response. One such key transcription factor that is known to modulate neuronal expression of PUMA and BIM in response to cytokine withdrawal is FOXO3a [34]. FOXO3a undergoes dephosphorylation and nuclear translocation in response to ER stress and this modification allows its transcriptional activation [33], though its downstream targets in response to ER stress are not well defined.

CHOP protein is composed of two known functional domains, an N-terminal transcriptional activation domain and a C-terminal basic-leucine zipper (bZIP) domain consisting of a basic amino-acid-rich DNA-binding region followed by a leucine zipper dimerization motif [22], [50]. Deletion mutant analysis of CHOP revealed that the bZIP domain is important for CHOP-induced apoptosis [25]. The high conservation (>90%) in the bZIP domain of the C/EBP members allows for formation of homodimers and heterodimers of the members [22]. Furthermore, CHOP can enhance the transcriptional activation of AP-1 by tethering to the AP-1 complex without direct binding of DNA [51]. A growing body of evidence also suggests that CHOP-mediated apoptosis occurs through non-transcriptional mechanisms such as protein–protein interactions [52], [53]. Thus, the regulation of CHOP-mediated apoptosis and its downstream targets is complex. According to a recent report, CHOP co-operates with AP-1 to mediate PUMA expression in a model of hepatic lipoapoptosis [54]. TRB3 a recently characterized downstream target of CHOP, was shown to directly interact with AKT and suppress the phosphorylation of this kinase in liver [55]. Our results indicate that a knockdown of CHOP expression in SH-SY5Y cells causes a decrease in PUMA protein and *Puma* mRNA levels in response to ER stress, suggesting that CHOP is a direct regulator of PUMA expression in response to ER stress. However, CHOP knowkdown does not completely restore PUMA expression to baseline levels suggesting that alternate transcription factors may also play a role. Our results indicate that CHOP knockdown inhibits the dephosphorylation of AKT and FOXO3a in response to ER stress in neuronal cells, suggesting that CHOP can feed into alternate signaling pathways such as the AKT/FOXO3a axis that may play a role in regulating expression of downstream pro-apoptotic genes *Puma* and *Bim*. The observed interaction between CHOP and FOXO3a suggests that CHOP regulation of downstream target genes such as Puma and Bim can also occur through modulation of the AKT/FOXO3a axis.

In summary, our study extends and further integrates present knowledge regarding the mechanisms linking ER stress, CHOP and the induction of BH3-only molecules in ER stress-induced apoptosis. Insights into the network regulating CHOP-mediated apoptosis will potentially provide a basis for new CHOP-targeted therapeutic approaches to ER stress-associated neurodegenerative diseases.

## References

[1] Schroder M, Kaufman RJ (2005). ER stress and the unfolded protein response. Mutat Res 569: 29–63.. S0027-5107(04)00371-9 [pii];10.1016/j.mrfmmm.2004.06.056 [doi].

[2] Schroder M (2008). Endoplasmic reticulum stress responses. Cell Mol Life Sci 65: 862–894.. 10.1007/s00018-007-7383-5 [doi].

[3] Wei MC, Zong WX, Cheng EH, Lindsten T, Panoutsakopoulou V, Ross AJ, Roth KA, MacGregor GR, Thompson CB, Korsmeyer SJ (2001). Proapoptotic BAX and BAK: a requisite gateway to mitochondrial dysfunction and death. Science 292: 727–730.. 10.1126/science.1059108 [doi];292/5517/727 [pii].

[4] Smith MI, Deshmukh M (2007). Endoplasmic reticulum stress-induced apoptosis requires bax for commitment and Apaf-1 for execution in primary neurons. Cell Death Differ 14: 1011–1019.. 4402089 [pii];10.1038/sj.cdd.4402089 [doi].

[5] Volchuk A, Ron D (2010). The endoplasmic reticulum stress response in the pancreatic beta-cell. Diabetes Obes Metab 12 Suppl 2: 48–57.. 10.1111/j.1463-1326.2010.01271.x [doi].

[6] Ogawa S, Kitao Y, Hori O (2007). Ischemia-induced neuronal cell death and stress response. Antioxid Redox Signal 9: 573–587.. 10.1089/ars.2006.1516 [doi].

[7] Doyle KM, Kennedy D, Gorman AM, Gupta S, Healy SJ, Samali A (2011). Unfolded proteins and endoplasmic reticulum stress in neurodegenerative disorders. J Cell Mol Med 15: 2025–2039.. 10.1111/j.1582-4934.2011.01374.x [doi].

[8] Adams JM, Cory S (2007). Bcl-2-regulated apoptosis: mechanism and therapeutic potential. Curr Opin Immunol 19: 488–496.. S0952-7915(07)00099-4 [pii];10.1016/j.coi.2007.05.004 [doi].

[9] Willis SN, Adams JM (2005). Life in the balance: how BH3-only proteins induce apoptosis. Curr Opin Cell Biol 17: 617–625.. S0955-0674(05)00150-X [pii];10.1016/j.ceb.2005.10.001 [doi].

[10] Bouillet P, Strasser A (2002). BH3-only proteins – evolutionarily conserved proapoptotic Bcl-2 family members essential for initiating programmed cell death.. J Cell Sci.

[11] Huang DC, Strasser A (2000). BH3-Only proteins-essential initiators of apoptotic cell death. Cell 103: 839–842.. S0092-8674(00)00187-2 [pii].

[12] Bouillet P, Purton JF, Godfrey DI, Zhang LC, Coultas L, Puthalakath H, Pellegrini M, Cory S, Adams JM, Strasser A (2002). BH3-only Bcl-2 family member Bim is required for apoptosis of autoreactive thymocytes. Nature 415: 922–926.. 10.1038/415922a [doi];415922a [pii].

[13] Reimertz C, Kogel D, Rami A, Chittenden T, Prehn JH (2003). Gene expression during ER stress-induced apoptosis in neurons: induction of the BH3-only protein Bbc3/PUMA and activation of the mitochondrial apoptosis pathway. J Cell Biol 162: 587–597.. 10.1083/jcb.200305149 [doi]; jcb.200305149 [pii].

[14] Puthalakath H, O'Reilly LA, Gunn P, Lee L, Kelly PN, Huntington ND, Hughes PD, Michalak EM, McKimm-Breschkin J, Motoyama N, Gotoh T, Akira S, Bouillet P, Strasser A (2007). ER stress triggers apoptosis by activating BH3-only protein Bim. Cell 129: 1337–1349.. S0092-8674(07)00537-5 [pii];10.1016/j.cell.2007.04.027 [doi].

[15] Li J, Lee B, Lee AS (2006). Endoplasmic reticulum stress-induced apoptosis: multiple pathways and activation of p53-up-regulated modulator of apoptosis (PUMA) and NOXA by p53. J Biol Chem 281: 7260–7270.. M509868200 [pii];10.1074/jbc.M509868200 [doi].

[16] Galehdar Z, Swan P, Fuerth B, Callaghan SM, Park DS, Cregan SP (2010). Neuronal apoptosis induced by endoplasmic reticulum stress is regulated by ATF4-CHOP-mediated induction of the Bcl-2 homology 3-only member PUMA. J Neurosci 30: 16938–16948.. 30/50/16938 [pii];10.1523/JNEUROSCI.1598-10.2010 [doi].

[17] Zinszner H, Kuroda M, Wang X, Batchvarova N, Lightfoot RT, Remotti H (1998). CHOP is implicated in programmed cell death in response to impaired function of the endoplasmic reticulum.. Genes Dev.

[18] Oyadomari S, Koizumi A, Takeda K, Gotoh T, Akira S, Araki E, Mori M (2002). Targeted disruption of the Chop gene delays endoplasmic reticulum stress-mediated diabetes. J Clin Invest 109: 525–532.. 10.1172/JCI14550 [doi].

[19] Ji C, Mehrian-Shai R, Chan C, Hsu YH, Kaplowitz N (2005). Role of CHOP in hepatic apoptosis in the murine model of intragastric ethanol feeding. Alcohol Clin Exp Res 29: 1496–1503.. 00000374-200508000-00017 [pii].

[20] Silva RM, Ries V, Oo TF, Yarygina O, Jackson-Lewis V (2005). CHOP/GADD153 is a mediator of apoptotic death in substantia nigra dopamine neurons in an in vivo neurotoxin model of parkinsonism. J Neurochem 95: 974–986.. JNC3428 [pii];10.1111/j.1471-4159.2005.03428.x [doi].

[21] Namba T, Tanaka K, Ito Y, Ishihara T, Hoshino T, Gotoh T, Endo M (2009). Positive role of CCAAT/enhancer-binding protein homologous protein, a transcription factor involved in the endoplasmic reticulum stress response in the development of colitis. Am J Pathol 174: 1786–1798.. S0002-9440(10)61036-X [pii];10.2353/ajpath.2009.080864 [doi].

[22] Ron D, Habener JF (1992). CHOP, a novel developmentally regulated nuclear protein that dimerizes with transcription factors C/EBP and LAP and functions as a dominant-negative inhibitor of gene transcription.. Genes Dev.

[23] Okada T, Yoshida H, Akazawa R, Negishi M, Mori K (2002). Distinct roles of activating transcription factor 6 (ATF6) and double-stranded RNA-activated protein kinase-like endoplasmic reticulum kinase (PERK) in transcription during the mammalian unfolded protein response. Biochem J 366: 585–594.. 10.1042/BJ20020391 [doi]; BJ20020391 [pii].

[24] Wang XZ, Ron D (1996). Stress-induced phosphorylation and activation of the transcription factor CHOP (GADD153) by p38 MAP Kinase.. Science.

[25] Maytin EV, Ubeda M, Lin JC, Habener JF (2001). Stress-inducible transcription factor CHOP/gadd153 induces apoptosis in mammalian cells via p38 kinase-dependent and -independent mechanisms. Exp Cell Res 267: 193–204.. 10.1006/excr.2001.5248 [doi]; S0014-4827(01)95248-6 [pii].

[26] Brunet A, Bonni A, Zigmond MJ, Lin MZ, Juo P, Hu LS (1999). Akt promotes cell survival by phosphorylating and inhibiting a Forkhead transcription factor. Cell 96: 857–868.. S0092-8674(00)80595-4 [pii].

[27] Brunet A, Datta SR, Greenberg ME (2001). Transcription-dependent and -independent control of neuronal survival by the PI3K-Akt signaling pathway. Curr Opin Neurobiol 11: 297–305.. S0959-4388(00)00211-7 [pii].

[28] del PL, Gonzalez VM, Hernandez R, Barr FG, Nunez G (1999). Regulation of the forkhead transcription factor FKHR, but not the PAX3-FKHR fusion protein, by the serine/threonine kinase Akt. Oncogene 18: 7328–7333.. 10.1038/sj.onc.1203159 [doi].

[29] Biggs WH, Meisenhelder J, Hunter T, Cavenee WK, Arden KC (1999). Protein kinase B/Akt-mediated phosphorylation promotes nuclear exclusion of the winged helix transcription factor FKHR1.. Proc Natl Acad Sci U S A.

[30] Kops GJ, Medema RH, Glassford J, Essers MA, Dijkers PF (2002). Control of cell cycle exit and entry by protein kinase B-regulated forkhead transcription factors.. Mol Cell Biol.

[31] Gilley J, Coffer PJ, Ham J (2003). FOXO transcription factors directly activate bim gene expression and promote apoptosis in sympathetic neurons. J Cell Biol 162: 613–622.. 10.1083/jcb.200303026 [doi]; jcb.200303026 [pii].

[32] Li D, Qu Y, Mao M, Zhang X, Li J, Ferriero D, Mu D (2009). Involvement of the PTEN-AKT-FOXO3a pathway in neuronal apoptosis in developing rat brain after hypoxia-ischemia. J Cereb Blood Flow Metab 29: 1903–1913.. jcbfm2009102 [pii];10.1038/jcbfm.2009.102 [doi].

[33] Zhu W, Bijur GN, Styles NA, Li X (2004). Regulation of FOXO3a by brain-derived neurotrophic factor in differentiated human SH-SY5Y neuroblastoma cells. Brain Res Mol Brain Res 126: 45–56.. 10.1016/j.molbrainres.2004.03.019 [doi]; S0169328X04001615 [pii].

[34] You H, Pellegrini M, Tsuchihara K, Yamamoto K, Hacker G, Erlacher M, Villunger A, Mak TW (2006). FOXO3a-dependent regulation of Puma in response to cytokine/growth factor withdrawal. J Exp Med 203: 1657–1663.. jem.20060353 [pii];10.1084/jem.20060353 [doi].

[35] Dudgeon C, Wang P, Sun X, Peng R (2010). PUMA induction by FoxO3a mediates the anticancer activities of the broad-range kinase inhibitor UCN-01. Mol Cancer Ther 9: 2893–2902.. 1535-7163.MCT-10-0635 [pii];10.1158/1535-7163.MCT-10-0635 [doi].

[36] Amente S, Zhang J, Lavadera ML, Lania L, Avvedimento EV, Majello B (2011). Myc and PI3K/AKT signaling cooperatively repress FOXO3a-dependent PUMA and GADD45a gene expression. Nucleic Acids Res 39: 9498–9507.. gkr638 [pii];10.1093/nar/gkr638 [doi].

[37] Villunger A, Michalak EM, Coultas L, Mullauer F, Bock G, Ausserlechner MJ, Adams JM, Strasser A (2003). p53- and drug-induced apoptotic responses mediated by BH3-only proteins puma and noxa. Science 302: 1036–1038.. 10.1126/science.1090072 [doi];1090072 [pii].

[38] Bouillet P, Metcalf D, Huang DC, Tarlinton DM, Kay TW, Kontgen F (1999). Proapoptotic Bcl-2 relative Bim required for certain apoptotic responses, leukocyte homeostasis, and to preclude autoimmunity. Science 286: 1735–1738.. 8026 [pii].

[39] Roth KA, Motoyama N, Loh DY (1996). Apoptosis of bcl-x-deficient telencephalic cells in vitro.. J Neurosci.

[40] Duksin D, Mahoney WC (1982). Relationship of the structure and biological activity of the natural homologues of tunicamycin.. J Biol Chem.

[41] Akhtar RS, Geng Y, Klocke BJ, Latham CB, Villunger A, Michalak EM (2006). BH3-only proapoptotic Bcl-2 family members Noxa and Puma mediate neural precursor cell death. J Neurosci 26: 7257–7264.. 26/27/7257 [pii];10.1523/JNEUROSCI.0196-06.2006 [doi].

[42] Nakano K, Vousden KH (2001). PUMA, a novel proapoptotic gene, is induced by p53. Mol Cell 7: 683–694.. S1097-2765(01)00214-3 [pii].

[43] Ogawa S, Kitao Y, Hori O (2007). Ischemia-induced neuronal cell death and stress response. Antioxid Redox Signal 9: 573–587.. 10.1089/ars.2006.1516 [doi].

[44] Reijonen S, Putkonen N, Norremolle A, Lindholm D, Korhonen L (2008). Inhibition of endoplasmic reticulum stress counteracts neuronal cell death and protein aggregation caused by N-terminal mutant huntingtin proteins. Exp Cell Res 314: 950–960.. S0014-4827(08)00002-5 [pii];10.1016/j.yexcr.2007.12.025 [doi].

[45] Kudo T, Katayama T, Imaizumi K, Yasuda Y, Yatera M, Okochi M, Tohyama M, Takeda M (2002). The unfolded protein response is involved in the pathology of Alzheimer's disease.. Ann N Y Acad Sci.

[46] Kim H, Tu HC, Ren D, Takeuchi O, Jeffers JR, Zambetti GP, Hsieh JJ, Cheng EH (2009). Stepwise activation of BAX and BAK by tBID, BIM, and PUMA initiates mitochondrial apoptosis. Mol Cell 36: 487–499.. S1097-2765(09)00690-X [pii];10.1016/j.molcel.2009.09.030 [doi].

[47] Wyttenbach A, Tolkovsky AM (2006). The BH3-only protein Puma is both necessary and sufficient for neuronal apoptosis induced by DNA damage in sympathetic neurons. J Neurochem 96: 1213–1226.. JNC3676 [pii];10.1111/j.1471-4159.2005.03676.x [doi].

[48] Ma Y, Brewer JW, Diehl JA, Hendershot LM (2002). Two distinct stress signaling pathways converge upon the CHOP promoter during the mammalian unfolded protein response. J Mol Biol 318: 1351–1365.. S0022-2836(02)00234-6 [pii].

[49] McCullough KD, Martindale JL, Klotz LO, Aw TY, Holbrook NJ (2001). Gadd153 sensitizes cells to endoplasmic reticulum stress by down-regulating Bcl2 and perturbing the cellular redox state. Mol Cell Biol 21: 1249–1259.. 10.1128/MCB.21.4.1249-1259.2001 [doi].

[50] Ubeda M, Wang XZ, Zinszner H, Wu I, Habener JF, Ron D (1996). Stress-induced binding of the transcriptional factor CHOP to a novel DNA control element.. Mol Cell Biol.

[51] Ubeda M, Vallejo M, Habener JF (1999). CHOP enhancement of gene transcription by interactions with Jun/Fos AP-1 complex proteins.. Mol Cell Biol.

[52] Wang H, Iakova P, Wilde M, Welm A, Goode T (2001). C/EBPalpha arrests cell proliferation through direct inhibition of Cdk2 and Cdk4. Mol Cell 8: 817–828.. S1097-2765(01)00366-5 [pii].

[53] Gotoh T, Terada K, Oyadomari S, Mori M (2004). hsp70-DnaJ chaperone pair prevents nitric oxide- and CHOP-induced apoptosis by inhibiting translocation of Bax to mitochondria. Cell Death Differ 11: 390-402.. 10.1038/sj.cdd.4401369 [doi];4401369 [pii].

[54] Cazanave SC, Elmi NA, Akazawa Y, Bronk SF, Mott JL, Gores GJ (2010). CHOP and AP-1 cooperatively mediate PUMA expression during lipoapoptosis. Am J Physiol Gastrointest Liver Physiol 299: G236-G243.. ajpgi.00091.2010 [pii];10.1152/ajpgi.00091.2010 [doi].

[55] Du K, Herzig S, Kulkarni RN, Montminy M (2003). TRB3: a tribbles homolog that inhibits Akt/PKB activation by insulin in liver. Science 300: 1574-1577.. 10.1126/science.1079817 [doi];300/5625/1574 [pii].

